# In this issue: Volume 114, Issue 9, September 2023

**DOI:** 10.1111/cas.15938

**Published:** 2023-08-24


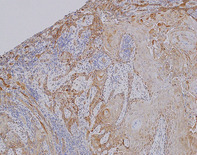


## Genetic and nongenetic mechanisms for colorectal cancer evolution


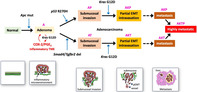


Colorectal cancer (CRC) originates in the large intestine and is a major cause of cancer‐related deaths worldwide. Its deadly nature stems from its ability to recur after treatment and spread to distant organs like the liver and lungs. Research reveals that CRC development occurs in stages, with genetic changes building up over time. However, scientists discovered no major differences in the genetic changes observed in the original tumor and the ones that had spread to other organs. This suggests that there might be other non‐genetic factors contributing to the ability of cancer cells to spread and develop in other organs, warranting a closer look at all possible factors responsible for disease development and progression.

This review article by researchers in Japan attempts to fill this knowledge gap.

The review suggests that there have been studies focusing on mice models with specific genetic mutations to unravel the genetic makeup of CRC. Through these studies, certain genes have been observed to play a vital role in the development and progression of the disease. One significant finding of recent research has been the identification of the primary mediators of CRC development, such as genetic alterations in the Wnt, MAPK/ERK, PI3K, p53, and TGF‐β pathways, which are important signaling pathways and genes that play critical roles in various cellular processes. Activation of Wnt signaling by APC mutation is critical in initiating CRC development, while p53 gain‐of‐function mutations, loss of TGF‐β signaling mutations, as well as KRAS activation mutations drive malignant progression.

Moreover, transplanting organoids bearing these mutations into the spleen of mice has been found to result in their spread to the liver. Studies have found the normal TP53 gene to be lost in half of these liver tumors; however, it was still retained in the tumors growing in the spleen. This suggests that losing the normal TP53 gene through a process called LOH (loss of heterozygosity) plays a significant role in driving CRC spread.

Further, research has also shown that genetic changes that occur during cancer development creates diverse cell clusters. These cell clusters exhibit different genetic makeup and contributes towards the spread of cancer. Cells in the tumor microenvironment, particularly cancer‐associated fibroblasts, play a crucial role in creating a specific environment called a ‘fibrotic niche’ in the new tumors that develop in distant parts of the body. This fibrotic niche supports the spread of the disease.

However, the authors of the article emphasize that cancer evolution is not only governed by genetic mechanisms but also by non‐genetic mechanisms. Positive selection supports tumor cells that spread and invade, while negative selection eliminates cells that lose their growth advantage despite acquiring genetic mutations. Thus, an understanding of the genetic and non‐genetic mechanisms that govern cancer cell diversity and evolution is necessary to provide more personalized and improved treatments for patients with advanced CRC.

https://onlinelibrary.wiley.com/doi/full/10.1111/CAS.15891 
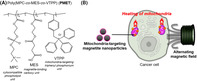


## Effective magnetic hyperthermia induced by mitochondria‐targeted nanoparticles modified with triphenylphosphonium‐containing phospholipid polymers

A promising treatment method for killing cancer cells with minimal side effects is the localized heating of tumors to temperatures above 42.5°C. This is achieved by supplying magnetite nanoparticles (MNPs), i.e., particles that exhibit magnetism, directly into cancer cells. Exposing the entire tumor area to an alternating magnetic field causes MNPs to heat up, due to Brownian and Néel relaxation—a phenomenon in which physical movements occur in nanoscales, causing generation of energy. The resultant heat stress causes tumors to become more vulnerable than normal tissues, since the former lack fully formed blood vessels to help with heat dissipation.

Owing to the excellent tissue permeability of alternating magnetic fields, this method works well even for deep‐seated tumors. Controlling the subcellular localization of nanoparticles and heating thermally sensitive regions within cancer cells is likely to further improve the anticancer efficacy of intracellular hyperthermia.

In this study, Kaneko et al. attempted to improve the therapeutic efficiency of this method by using mitochondria‐targeting MNPs. These were prepared by modifying carboxyl phospholipid polymers containing triphenylphosphonium moieties, which could guide these MNPs directly into mitochondria. The team confirmed mitochondrial localization through transmission electron microscopy observations of mouse colon cancer cells, that had been treated with polymer‐modified MNPs.

Next, Kaneko et al. tested the efficacy of these polymer‐modified MNPs on both—an *in vivo* mouse model of colon cancer, as well as cultured mouse colon cancer cells. Their results revealed that the therapeutic effects of polymer‐modified MNPs were enhanced by the addition of triphenylphosphonium.

These findings indicate that localized heating of mitochondria within cancer cells can greatly improve the efficacy of the magnetic hyperthermia process. This approach is likely to pave the way for the further refinement of therapeutic strategies using magnetic hyperthermia, and for developing new, innovative strategies involving MNP surface design.

https://onlinelibrary.wiley.com/doi/full/10.1111/CAS.15895 
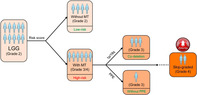


## Characterization and clinical implications of different malignant transformation patterns in diffuse low‐grade gliomas

Low‐grade glioma (LGG) is an early‐stage (stage 1 or 2) cancer of the brain cells that can be easily diagnosed due to its distinct characteristics. However, it has a tendency for malignant transformation (MT)—the ability to transform into its aggressive version, high‐grade glioma (HGG), a stage 3 or 4 cancer—despite aggressive treatment. It is thus critical to identify certain red flags that can help in the early detection of MT, in order to chalk out the right course of treatment for a patient with LGG. While previous studies have focused on using imaging techniques such as magnetic resonance imaging (MRI) to identify the predictive signs of MT, this approach has proven only limitedly successful.

In this issue, Jiang *et al*. attempted to address these challenges. The authors reviewed data from 229 patients with LGG in order to identify the different patterns associated with MT. They used various molecular, histopathological, and neuroimaging techniques to analyze different characteristics of an LGG. Based on these, they classified the patients into three categories: ‘group 2‐3’ (patients who transformed from glioma stage 2 to 3), ‘group 2‐4’ (patients who transformed from glioma stage 2 to 4), and ‘group 2‐2’ (patients in whom the glioma did not undergo MT).

Patients from group 2‐2 had the longest survival while group 2‐4 had the shortest. Patients with a lower score on the Karnofsky performance scale (a tool that assesses the seriousness of an illness, with a lower score indicating more serious illness), bigger tumor sizes, smaller sections of tissue removed during surgery, and faster growth of cancer cells were more likely to experience MT. Moreover, patients with a specific genetic marker, ‘1p/19q codeletion’, were less likely to undergo MT.

Based on these observations, the researchers developed a special prediction model, a nomogram, that could accurately predict the likelihood of MT in patients with LGG. Further, they also introduced a novel term, ‘skip‐graded MT’, which identifies an LGG that has transformed directly from stage 2 to stage 4. These results have not only opened the door to a better understanding of gliomas but have also paved the way for their accurate diagnosis and timely treatment.

https://onlinelibrary.wiley.com/doi/full/10.1111/CAS.15889

